# Innovative application of Near Field Communication technology in dental prostheses and dental appliances

**DOI:** 10.1111/jopr.70104

**Published:** 2026-01-23

**Authors:** Sally Roushdy, Tommy Zhu, Scott Hendricks, Tanveer Vasdev, Galen Schneider

**Affiliations:** ^1^ Department of Prosthodontics, College of Dentistry and Dental Clinics University of Iowa Iowa City Iowa USA; ^2^ College of Dentistry and Dental Clinics University of Iowa Iowa City Iowa USA

**Keywords:** continuity of patient care, dental appliances, dental prostheses, electronic health record, Near Field Communication (NFC) technology, patient identification

## Abstract

Marking dental prostheses and appliances enhances patient identification and the continuity of care. This article presents a technique using the Near Field Communication (NFC) technology. The low‐cost miniature NFC chip, programmable through smartphones, can be embedded into dental prostheses or appliances to securely store and retrieve the patient's medical and dental information. This method allows immediate access to essential data in clinical and emergency situations, improves documentation, and provides a versatile solution for contemporary prosthodontic practice.

The marking of dental prostheses and appliances provides a practical means of identification in dental practices and facilitates the recovery of lost or misplaced items in hospitals and residential care facilities. Various methods of denture identification techniques have been reported in the literature, including surface marking and inclusion methods.^1^, ^2^ Among the inclusion methods, the use of bulky radio frequency identification (RFID) tags and quick response (QR) codes has been reported.^3^, ^4^, ^5^, ^6^ However, these tags and codes present limitations when applied to complex prosthetic designs or appliances with restricted space, such as hybrid prostheses and surgical guides, where their incorporation can compromise esthetics and structural integrity. The low‐cost miniature Near Field Communication (NFC) chips offer a promising alternative. The NFC technology is a low‐power wireless communication method used in contemporary daily life, enabling data exchange through close‐range contact.^7^ A smartphone is required to activate and program the NFC chip using an NFC programmable application. Contemporary smartphones have a built‐in NFC functionality and can read the NFC chips without the need for an additional application. However, older models may require an NFC reader and writer application.

Incorporating a miniature NFC chip into dental prostheses or appliances allows clinicians to store and retrieve essential patient information, such as medical and dental history, treatment records, blood type, implant specifications, and surgical reports. The chip can also be adapted for use with a wide range of dental prostheses and appliances, enhancing its versatility across diverse clinical applications.

The steps for programming and embedding the NFC chip into dental prostheses and appliances are described below.

## TECHNIQUE

### Programming the NFC chip

**FIGURE 1:**
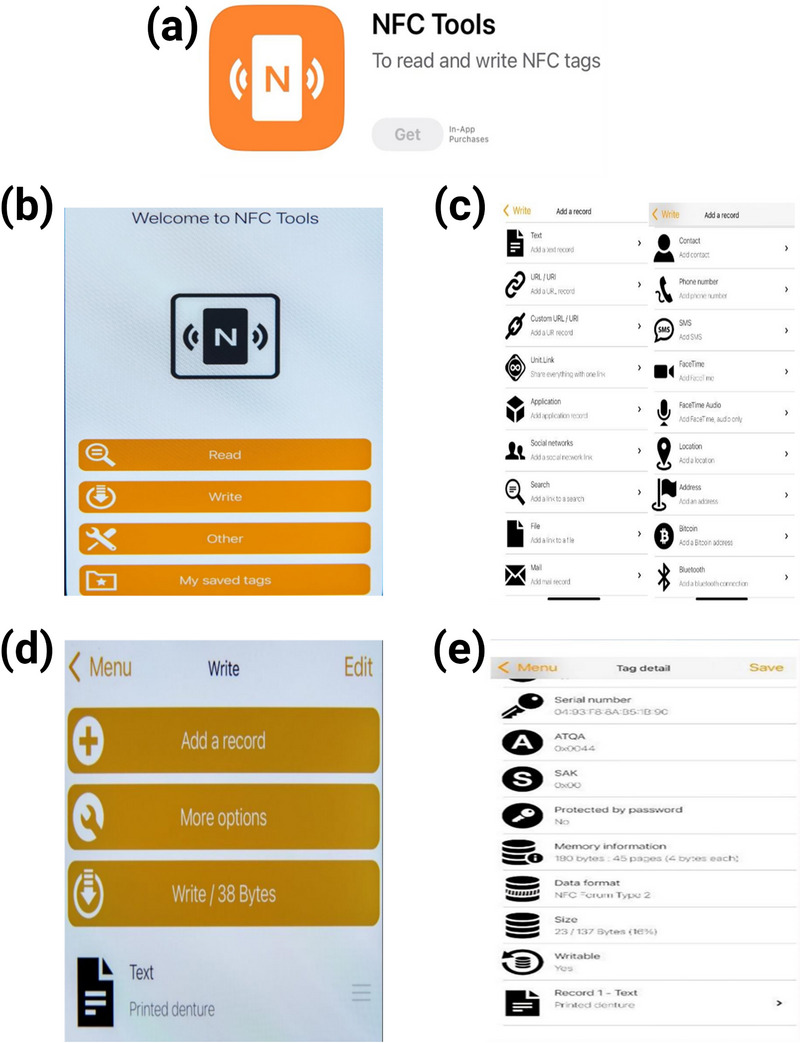
(a) The NFC Tools application. (b) Select “Write”. (c) The NFC application list of records. (d) “Printed denture” as a stored text record. (e) Read stored text record.

**FIGURE 2:**
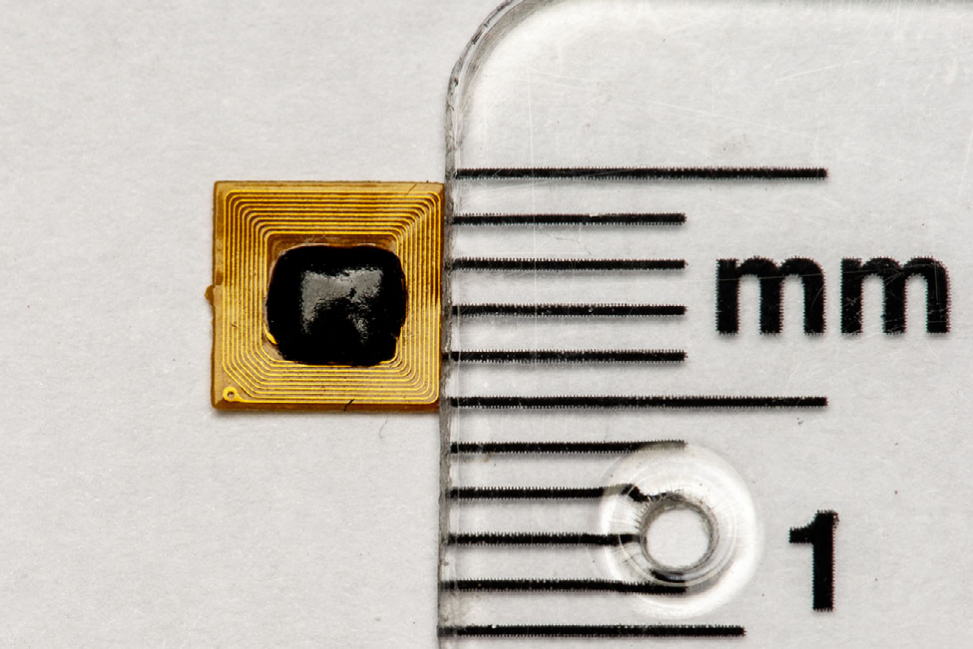
The NFC chip.

1. Download and install the NFC programmable application (NFC Tools; WAKDEV, Fontaine‐les‐Dijon, France) (Figure 1a).
2. Open the application and select **Write** (Figure 1b).
3. Tap **Add a record** and select the desired record type from the list of options (Figure 1c).
4. Hold the NFC chip (NFC RFID 213 chip; Yanzeo, Guangzhou, China) (Figure 2) near the phone and tap **Write** to store the text (Figure 1d).
5. Tap **Read** and the stored text will automatically open (Figure 1e).

### NFC Chip embedding technique

**3D‐Printed Surgical Template**

**FIGURE 3:**
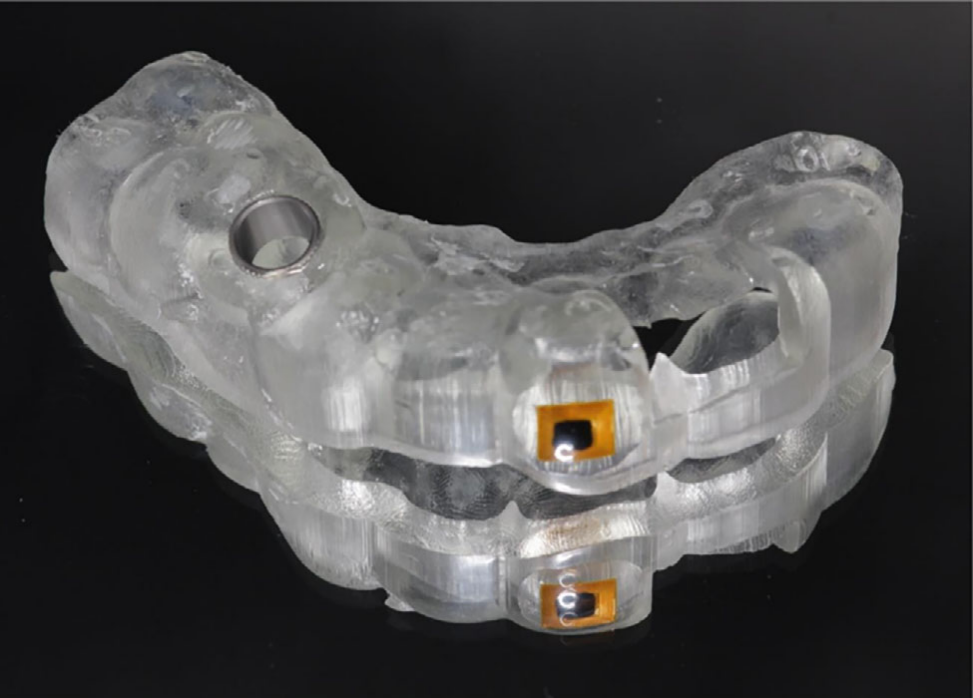
NFC chip embedded in a 3D‐printed surgical template.

**FIGURE 4:**
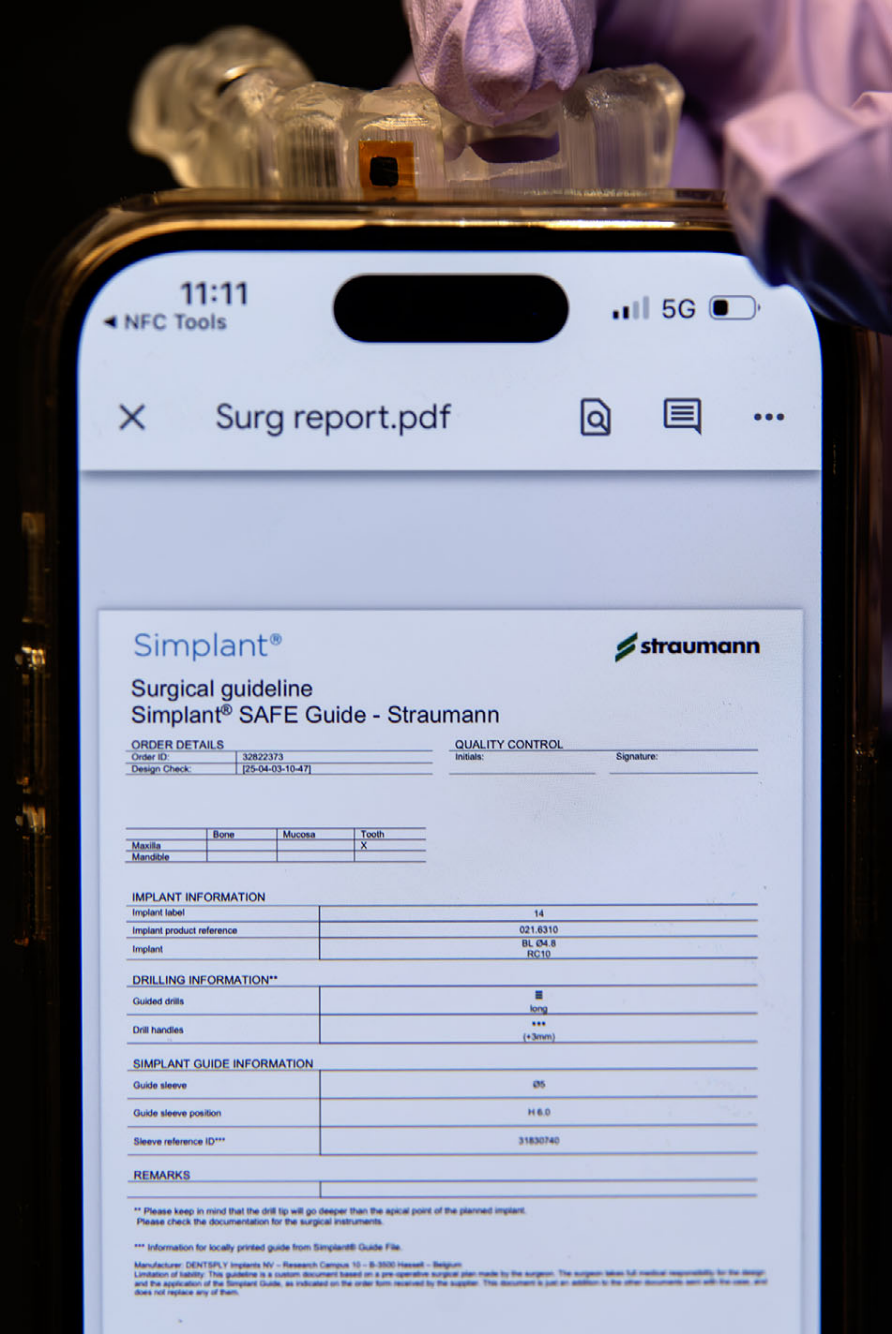
Information retrieved by a smartphone. The NFC chip contained a URL record that opened a surgical report in PDF format.

1. Place the NFC chip (NFC RFID 213 chip; Yanzeo, Guangzhou, China) on the outer surface of a 3D‐printed surgical template using its integrated self‐adhesive backing.
2. Apply light‐polymerizing resin (Surgical Guide 3; SprintRay Inc., Los Angeles, CA) over the NFC chip to seal it.
3. Light‐polymerize the resin for 5 minutes in a light‐polymerizing unit (Procure 2; SprintRay Inc., Los Angeles, CA).
4. Smooth the surface with a rubber wheel and polish to a high shine (Figure 3).
5. Scan the embedded NFC chip with a smartphone to retrieve the stored information (Figure 4).

**Zirconia Complete‐Arch Implant‐Supported Fixed Dental Prosthesis**

**FIGURE 5:**
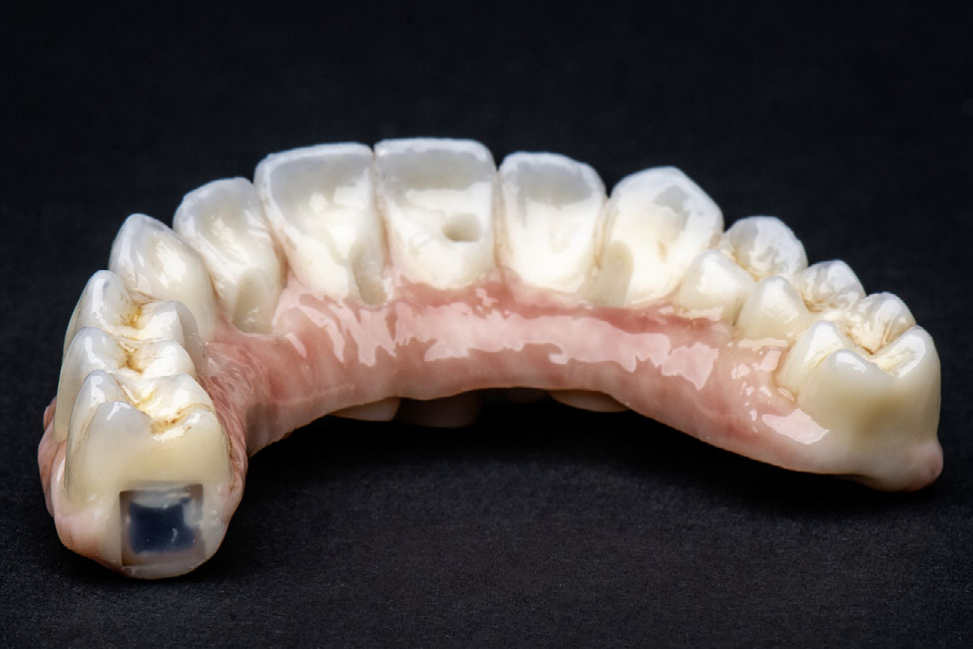
NFC chip embedded in a zirconia complete‐arch implant‐supported fixed dental prosthesis.

**FIGURE 6:**
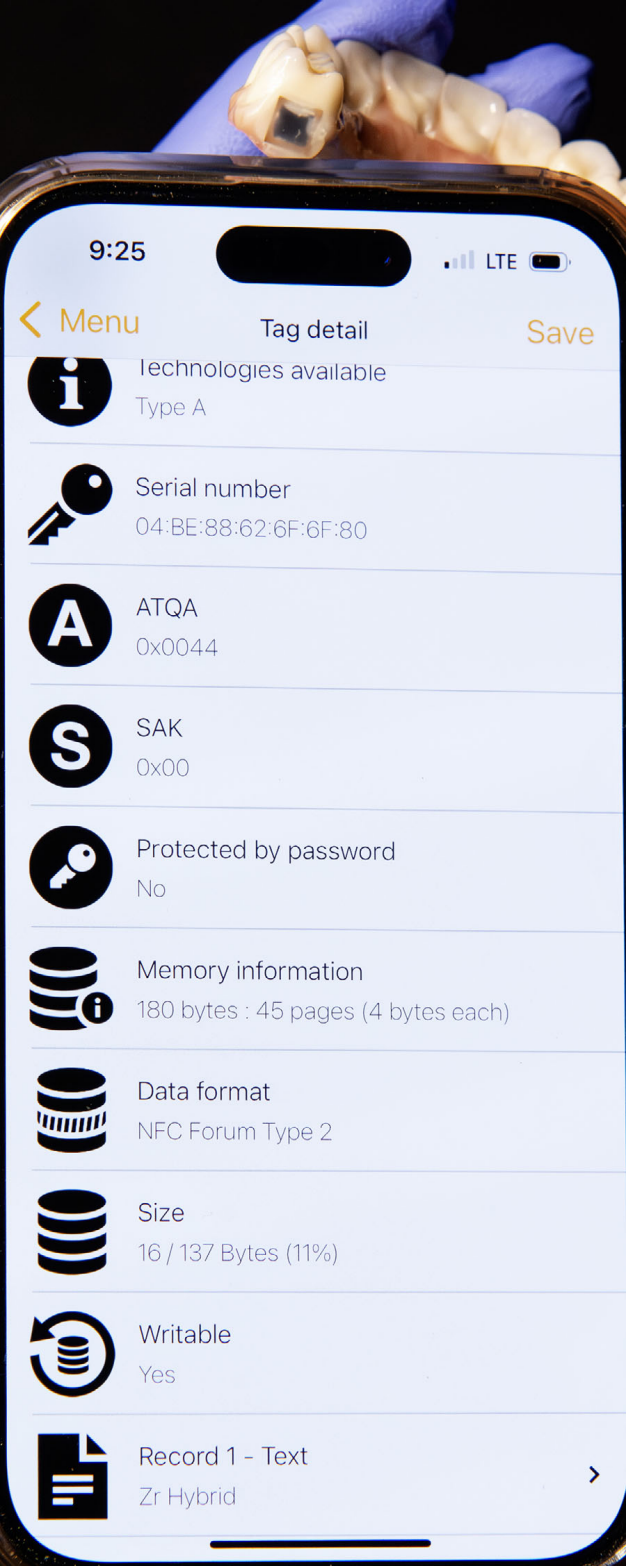
Information retrieved by a smartphone. The NFC chip was written with the text record: “Zr Hybrid.” Additional information can be added as needed.

1. Mark the outline of the NFC chip on the distal wall of the most terminal tooth during the pre‐sintering stage of a zirconia complete‐arch implant‐supported fixed dental prosthesis.
2. Cut a recess 1 mm deep within the marked outline using a coarse diamond bur.
3. Sinter the prosthesis at 1500°C for 8 hours.
4. Sandblast the trough area with 40‐micron aluminum oxide particles once it has cooled to room temperature, then steam‐clean and air‐dry.
5. Apply adhesive (Monobond Plus; Ivoclar Vivadent AG, Schaan, Liechtenstein) to the trough for 60 seconds, and dry with oil‐free air.
6. Place the NFC chip (NFC RFID 213 chip; Yanzeo, Guangzhou, China) into the trough and cover it with a clear cement (Multilink Clear Automix; Ivoclar Vivadent, Schaan, Liechtenstein).
7. Place the prosthesis in a light‐polymerizing unit (Labolight Duo; GC America Inc., Alsip, IL) for 1 minute.
8. Smooth the surface with a rubber wheel and apply a protective coat of clear glaze (HV Optiglaze; GC America Inc., Alsip, IL) and polymerize it for 1 minute in the light‐polymerizing unit (Figure 5).
9. Scan the embedded NFC chip with a smartphone to retrieve the stored information (Figure 6).

## DISCUSSION

**FIGURE 7:**
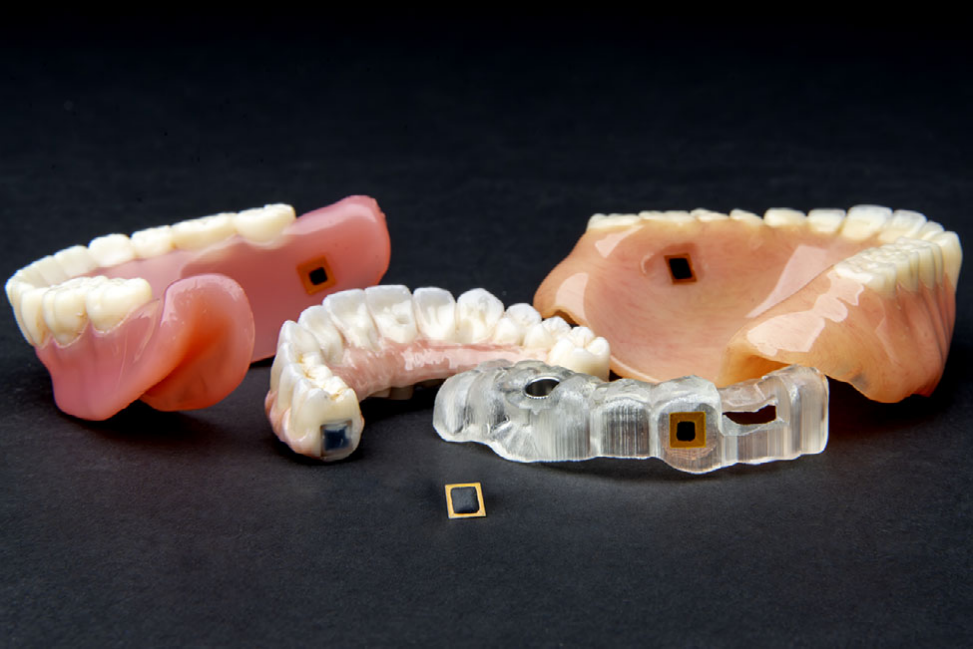
Several variations of the technique using an NFC chip.

This article highlights the innovative application of NFC technology in dental prostheses and appliances (Figure 7). Programmed NFC chips can be embedded in dental prostheses or appliances using simple and reliable techniques, enabling easy retrieval of stored information using smartphones. A variety of user‐friendly applications are currently available that enable NFC chipprogramming and provide interactive functions, such as linking to websites, educational videos, or other digital content, thereby serving as a novel communication tool.

Security features greatly vary among NFC applications and NFC chip types, and clinicians can select and implement the appropriate security measures based on the specific clinical context and their professional judgment. Password encryption further safeguards the stored information while allowing updates, flexibility, and security. Within the NFC Tools application, the password management option can be accessed through the “Other” menu (Figure 1b), which allows the user to select either the “Set password” or “Remove password” option.

In accordance with HIPAA regulations, patients can provide informed consent prior to embedding personal, medical, or dental information into the NFC chip, ensuring both ethical and legal use of patient data.

The low‐cost NFC chip (NFC RFID; Yanzeo, Guangzhou, China) used in this study was extremely small, measuring approximately 5 mm × 5 mm in size with a thickness of 0.1 mm at the center and 0.5 mm at the borders (Figure 2). It was equipped with a self‐adhesive backing to facilitate its incorporation. The chip had a maximum gain value of 1.5 dBm and an induction distance of 8–20 mm, depending on the device reader. The operating temperature of this chip ranged from ‐20°C to 85°C.

Further research is needed to evaluate the effect of humidity and thermal fluctuations in the oral environment on the performance of the embedded NFC chips.

## CONCLUSIONS

This article describes a practical and efficient technique in which a low‐cost miniature NFC chip is programmed and embedded in the dental prosthesis or appliance, allowing clinicians to access essential patientinformation using a smartphone. The NFC chip provides an esthetically pleasing method for storing and retrieving electronic health and dental records, thereby enhancing continuity of care and supporting clinical decision‐making. The technique enables reliable data insertion and retrieval across a wide variety of prosthetic designs.

## CONFLICT OF INTEREST STATEMENT

The authors declare no conflicts of interest.
